# Different temporal relationship between sex hormones and sleep status in midlife women: a longitudinal cohort study

**DOI:** 10.1093/sexmed/qfaf009

**Published:** 2025-03-02

**Authors:** Dongyuan Ma, Tong Zhang

**Affiliations:** The Acumox and Tuina College, Shandong University of Traditional Chinese Medicine, Jinan 250013, China; The Acumox and Tuina College, Shandong University of Traditional Chinese Medicine, Jinan 250013, China

**Keywords:** Temporal relationship, Sex hormone, Sleep, Midlife women

## Abstract

**Background:**

Fluctuation in sex hormones and the occurrence of sleep disturbance are 2 major health challenges among midlife women. However, the temporal relationship between them remains unclear.

**Methods:**

This study included 2488 females (mean ± SD age, 49.0 ± 2.70 years) with an average follow-up of 6.95 years. We constructed a composite score by summing items related to sleep problems to reflect the comprehensive sleep status of the participants in the Study of Women’s Health Across the Nation. Cross-lagged path analysis was used to examine the temporal relationship between sex hormones and sleep status. Sensitivity analyses were conducted in nonoverweight and overweight groups and adjusted for vasomotor symptoms in the main model.

**Aim:**

In this study, we aimed to examine the temporal relationship between sex hormones and sleep status in midlife women using cross-lagged path analysis.

**Outcomes:**

The primary outcomes included results of the cross-lagged path analysis between sex hormones and sleep status.

**Results:**

After adjusting for age, race, income, menopausal status, body mass index, hormone therapy use, smoking, and drinking, the cross-lagged path coefficients from baseline follicle-stimulating hormone (FSH) and estradiol (E2) to follow-up sleep status were 0.054 (*P* = .017) and –0.054 (*P* = .016), respectively. The path coefficient from baseline sleep to follow-up dehydroepiandrosterone sulfate (DHAS) was 0.042 (*P* = .017). The path coefficients between testosterone and sleep were not statistically significant. In the nonoverweight group, the patterns of the temporal relationship between sex hormones and sleep were the same as the total sample, and the point estimates were larger. However, the temporal relationships in the overweight group were nonsignificant. After adjustment for vasomotor symptoms in the main model, results were basically consistent.

**Clinical Implications:**

Given the temporal relationship between sex hormones and sleep, our findings will provide scientific perspectives to benefit health management in the transition of menopause.

**Strengths and Limitations:**

This study used a longitudinal theoretical model to distinguish the temporal relationship between sex hormones and sleep status in midlife women. Limitations include limited causal evidence in observational studies, unknown confounders, and careful extrapolation.

**Conclusion:**

There were distinct patterns in the unidirectional temporal relationship between (1) FSH, E2, and DHAS and (2) sleep. Changes in FSH and E2 occurred earlier than the change of sleep, while the change of DHAS was later. In contrast, there was no temporal relationship between testosterone and sleep.

## Introduction

Fluctuations in sex hormones and the increase in prevalence of sleep disturbances are two of the most common health issues in midlife women during menopause. Menopause is associated with significant changes in the hormonal environment, with an increase in follicle-stimulating hormone (FSH) mainly due to the reduced activity in the negative feedback system as ovarian follicle reserve declines. During the early menopausal transition, serum levels of estradiol (E2) are unstable and then drop to low levels after menopause.^1^ Serum androgen levels such as total testosterone (T) and dehydroepiandrosterone sulfate (DHAS) have been shown to decrease with age.^2^^,^^3^ Meanwhile, sleep disorders accompany menopause and aging, and women are more likely to experience them than men, with incidence ranges from 16% to 47% in perimenopause and 35% to 60% after menopause.^4^ Disordered sleep–associated medical consequences are a substantial financial burden on the health care system. For instance, annual costs of treating moderate to severe disordered sleep and its consequences in the United States are $165 billion, significantly greater than that for other noncommunicable diseases.^5^ Numerous studies showed that sex steroid hormones were synchronized with circadian rhythms and closely related to sleep.^6-9^ Estrogen replacement therapy could improve sleep quality effectively in females.^10^

The relationships between sex hormones and sleep are rather complex. For example, a comparative study found that increased FSH levels were associated with difficulty falling asleep and poorer subjective sleep quality.^11^ One possible explanation was that fluctuations in FSH may exacerbate vasomotor symptoms (VMSs), leading to facial and anterior chest flushing accompanied by night sweats, thereby affecting sleep quality.^12^ However, Touzet et al reported that the level of FSH was higher in long-time sleepers than in short-time sleepers.^9^ Similarly, Kravitz et al found that reduced E2 levels were associated with higher risk of difficulties falling asleep and staying asleep, but the authors also noted that baseline E2 was modestly and negatively associated with sleep quality in another study.^13^^,^^14^ To date, there are few studies focused on the effects of androgens on sleep in women.^15^ Moreover, the temporal relationship between sex hormones and sleep status in midlife women remains unclear.

Understanding the temporal patterns between hormones and sleep is vital for developing targeted strategies and interventions that could benefit women in the transition of menopause. Based on the cross-lagged panel design within a structural equation modeling framework, the cross-lagged path analysis model is an effective statistical method to explore the temporal relationship between variables with ≥2 panel data in a longitudinal cohort.^16^^,^^17^ This method can take the time stability of variables into consideration by including autocorrelation coefficients in the model and distinguish the temporal relationship between variables by comparing the cross-lagged path coefficients.^18^ The cross-lagged path model has recently been widely used in longitudinal cohort studies to explore the temporal relationship between chronic risk factors in psychology and biomedicine.^19^^,^^20^

In this study, we aimed to examine the temporal relationship between sex hormones and sleep status in midlife women using cross-lagged path analysis to provide a scientific perspective to benefit the health management in the transition of menopause.

## Methods

The data that support the findings of this study are publicly available at https://www.swanstudy.org.

### Participants

The Study of Women’s Health Across the Nation (SWAN) is a multiethnic, multicenter, community-based longitudinal study designed to characterize the psychological and biological changes of midlife women in the United States. In 1996, a total of 3302 premenopausal women were recruited from 7 sites across the nation for baseline examination: Boston, Chicago, Detroit, Los Angeles, Newark, Oakland, and Pittsburgh. The follow-up of this cohort is ongoing, and the data of baseline and the first 10 visits have been made public. Study protocols were approved by the institutional review board at each site, and all participants provided written informed consent at each study visit. A detailed description of the profile of this cohort can be found in a previous publication.^21^

**Figure 1:**
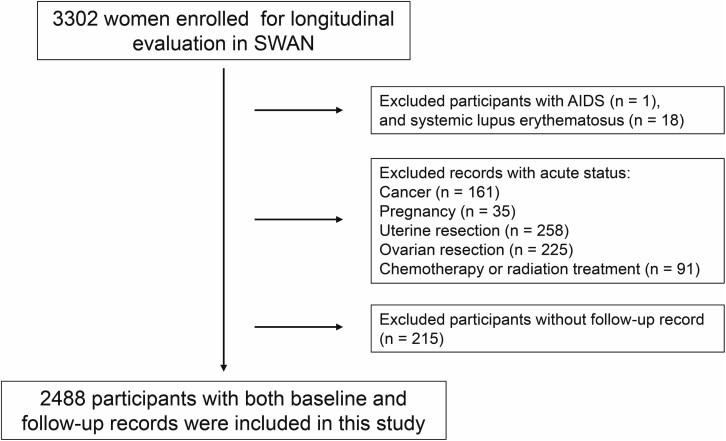
Flowchart of study participant selection and exclusion.

In the current study, to ensure a sufficient sample size and follow-up time, we selected the first 3 visits as baseline and the last 3 visits as follow-up from the cohort based on data availability of the interview information related to sleep status. Participants diagnosed with AIDS (n = 1) and systemic lupus erythematosus (n = 18) were excluded. Meanwhile, records indicating acute status during baseline and follow-up were also left out, including cancer (n = 161), pregnancy (n = 35), uterine resection (n = 258), ovarian resection (n = 225), and chemotherapy or radiation treatment (n = 91). Finally, 2488 females (mean age, 49.0 years at baseline) with baseline and follow-up records were included in this study, with an average 6.95 years follow-up (Figure 1). The first record of these participants at baseline and the last record at follow-up were extracted as our study cohort.

### Measurement of sleep status

Participants were interviewed by asking about sleep problems at each annual assessment: “frequency of feeling restless (almost always, often, sometimes, or almost never),” “frequency of trouble falling asleep,” “waking up several times a night,” “waking up earlier than planned and unable to fall asleep again (5 or more times a week, 3 or 4 times a week, 1 or 2 times a week, less than once a week, or not in the past 2 weeks),” and “how to rate your sleep quality overall during the past month (very bad, fairly bad, fairly good, very good).” We recoded these variables to make a higher score represent better sleep (Table S1), and we constructed a composite score by summing them to reflect a comprehensive sleep status of the participants.

### Measurement of sex hormone

Participants were required to collect venous blood in the morning after a fasting period of no fewer than 10 hours during days 2 to 5 of the follicular phase of the menstrual cycle. If specimens were not available after 2 samples in 2 to 5 days or if participants stopped menstruating or lacked a follicular phase sample, fasting blood specimens were randomly collected within 90 days of the annual evaluation. Blood samples were frozen in –80 °C after centrifugation and shipped on dry ice to the laboratory.

Sex hormones were measured by the Automated Chemiluminescence System–180 (ACS-180) analyzer at a specialized laboratory. Serum concentration of FSH was measured by a 2-site chemiluminometric immunoassay; serum concentration of E2 was measured with a modified offline ACS-180 (estradiol 6) immunoassay; and serum concentrations of DHAS and T were measured with a chemiluminescent assay based on a modified ACS-180 developed in the CLASS Laboratory. Duplicate E2 assays were performed to report the arithmetic mean for each participant as the result; all other assays were single determinations.

### Covariates

Information on age, race and ethnicity, body mass index (BMI), income, menopausal status, hormone therapy (HT) use, smoking and drinking, and VMSs was collected at each visit. BMI was calculated as weight in kilograms divided by height in meters squared. Self-reported income was defined as ≤$19 999, $20 000 to $49 999, $50 000 to $99 999, and ≥$100 000 per year. Menopausal status was determined by bleeding criteria. Receiving any of the following treatments was defined as HT use: birth control pills, estrogen pills (eg, Premarin, Estrace, Ogen), estrogen by injection or patch (eg, Estraderm), hormone combination therapy, and progestin pills (eg, Provera). Smokers and drinkers were defined as current smoking and drinking at least once a week. Additionally, VMS was defined as having in the past 2 weeks at least 1 symptom of hot flashes, cold sweats, or night sweats.

### Statistical analysis

Summary statistics of study variables were compared by mean (SD) and number (percentage) between baseline and follow-up. To reduce the skewness, all hormone values were log transformed. In the current study, cross-lagged path analysis was used to explore the temporal relationship between sex hormones and sleep status.

As a method that could simultaneously examine the temporal sequence of intercorrelated variables under a longitudinal framework, cross-lagged path analysis has been widely used in the fields of psychology and biomedicine (Figure S1).^18^ This model incorporates the temporal stability of the variable by incorporating the autocorrelation coefficient and assumes consistency within the population.^16^ To account for the impact of potential confounding factors, values of indicators at baseline and follow-up were adjusted separately for corresponding age, race, income, menopausal status, BMI, HT use, smoking, and drinking by regression residual models in the main analysis. Then the residuals were standardized through *Z* transformation^22^ to obtain the standardized regression coefficients. The cross-lagged path coefficients β_1_ and β_2_ represent the association between baseline *Y*_1_ and follow-up *X*_2_ and the association between baseline *X*_1_ and follow-up *Y*_2_, respectively. The significance of path coefficient β_1_ or β_2_ indicates a clear temporal relationship. The synchronous correlation *r*_1_ describes the correlation between *X*_1_ and *Y*_1_ at baseline, and the tracking correlations *r*_2_ and *r*_3_ represent the autocorrelation coefficients of *X* and *Y* between baseline and follow-up. The cross-lagged path models were fitted by structural equation modeling with the R package *Lavaan* (version 0.6-15). Consistent with a previous study, we used the root mean square residual (RMR) and comparative fit index (CFI) to assess the validity of model fitting,^23^ with RMR <0.05 and CFI >0.90 suggesting a good fit.

Sensitivity analyses were conducted in nonoverweight and overweight groups with adjustment for age, race, income, menopausal status, HT use, smoking, and drinking. According to the World Health Organization classification of overweight and obesity in adults, participants with a BMI ≤25 were defined as nonoverweight, and those with a BMI >25 were defined as overweight.^24^ Additionally, we adjusted for VMSs in the main model and performed another sensitivity analysis to validate the temporal relationship shown in the main analysis. All analyses in this study were conducted in R version 4.1.3. Two-sided *P* < .05 was considered statistically significant.

## Results

A total of 2488 females were included in the current study (Figure 1). Characteristics of the participants at baseline and follow-up are summarized in Table 1. The mean ± SD age at baseline was 49.0 ± 2.70 years and the average follow-up was 6.95 years. The study sample consisted of 48.9% Caucasians, 27.3% African Americans, 10.3% Japanese, 8.76% Chinese, and 4.7% Hispanic. According to menstrual bleeding criteria, 11.9% of the total sample was premenopausal at baseline, 61.9% early or late perimenopausal, and 12.8% natural postmenopausal. By the time of follow-up, 75.2% was postmenopausal. BMI and serum concentrations of FSH and T were significantly higher at follow-up than baseline, while E2, DHAS, score of sleep status, and proportion of HT use were significantly lower.

**Table 1:** Characteristics of the study cohort at baseline and follow-up.

**Variable**	**Baseline (N = 2488)**	**Follow-up (N = 2488)**	***P* value** ^**a**^
**Mean (SD)**			
Age, y	49.0 (2.70)	55.9 (2.73)	<.001
BMI, kg/m^2^	28.6 (7.13)	29.1 (7.05)	.018
FSH, mIU/mL	47.2 (46.1)	108.9 (55.0)	<.001
E2, pg/mL	53.3 (41.2)	26.7 (25.8)	<.001
DHAS, μg/dL	134.6 (82.2)	120.1 (75.0)	<.001
T, ng/dL	37.3 (19.8)	44.2 (40.5)	<.001
Score of sleep status	16.9 (5.21)	14.3 (7.22)	<.001
**No. (%)**			
Income, $			<.001
≤19 999	210 (9.23)	175 (8.78)	
20 000-49 999	619 (27.2)	524 (26.3)	
50 000-99 999	915 (40.2)	446 (22.4)	
≥100 000	532 (23.4)	847 (42.5)	
Status			<.001
Premenopause	297 (11.9)	21 (0.844)	
Early or late perimenopause	1542 (61.9)	492 (19.8)	
Natural postmenopause	319 (12.8)	1870 (75.2)	
Race			—
Black/African American	678 (27.3)	678 (27.3)	
Chinese/Chinese American	218 (8.76)	218 (8.76)	
Japanese/Japanese American	257 (10.3)	257 (10.3)	
Caucasian/White non-Hispanic	1218 (48.9)	1218 (48.9)	
Hispanic	117 (4.70)	117 (4.70)	
HT use	362 (14.6)	177 (7.11)	<.001
Smoker	327 (13.1)	283 (11.4)	.063
Drinker	715 (28.7)	724 (29.1)	.802
Hot flashes			<.001
Not at all	1402 (58.2)	1239 (51.2)	
1-5 d	632 (26.2)	640 (26.4)	
6-8 d	152 (6.30)	136 (5.62)	
9-13 d	96 (3.98)	95 (3.92)	
Every day	129 (5.35)	311 (12.8)	
Cold sweats			<.001
Not at all	2083 (87.4)	2034 (88.2)	
1-5 d	222 (9.32)	183 (7.93)	
6-8 d	33 (1.39)	24 (1.04)	
9-13 d	17 (0.714)	14 (0.607)	
Every day	26 (1.09)	52 (2.25)	
Night sweats			<.001
Not at all	1526 (63.5)	1541 (63.6)	
1-5 d	623 (25.9)	544 (22.5)	
6-8 d	113 (4.69)	95 (3.92)	
9-13 d	66 (2.74)	66 (2.72)	
Every day	77 (3.20)	177 (7.31)	

Abbreviations: BMI, body mass index; DHAS, dehydroepiandrosterone sulfate; E2, estradiol; FSH, follicle-stimulating hormone; HT, hormone therapy; T, testosterone.

^a^ *P* value for the difference of variables between baseline and follow-up.

**Figure 2:**
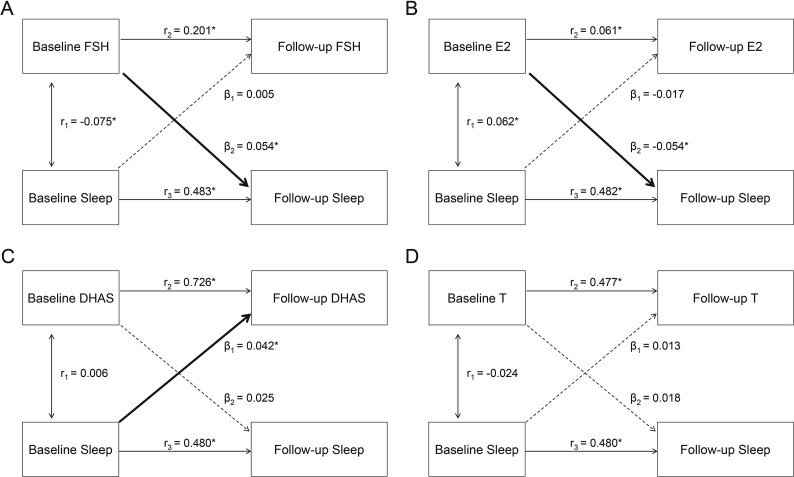
Cross-lagged path model between sex hormones and sleep status, adjusted for age, race, income, status, BMI, HT use, smoking, and drinking. β_1_ and β_2_, cross-lagged path coefficients; *r*_1_, synchronous correlations; *r*_2_ and *r*_3_, tracking correlations. ^*^P < .05. BMI, body mass index; DHAS, dehydroepiandrosterone sulfate; E2, estradiol; FSH, follicle-stimulating hormone; HT, hormone therapy; T, testosterone.

Figure 2 and Table S2 show the cross-lagged path model between sex hormones and the sleep status score (termed “sleep” in the following) after adjusting for age, race, income, menopausal status, BMI, HT use, smoking, and drinking. The synchronous correlation between baseline FSH and baseline sleep was –0.075 (95% CI, –0.125 to –0.024; *P* = .004). The autocorrelation coefficients of FSH and sleep were 0.201 (95% CI, 0.152-0.250) and 0.483 (95% CI, 0.440-0.527), respectively. The path coefficient from baseline FSH to follow-up sleep was 0.054 (95% CI, 0.010-0.098; *P* = .017) and that of baseline sleep to follow-up FSH was 0.005 (95% CI, –0.044 to 0.054; *P* = .842). The model evaluation indexes RMR and CFI were 0.009 and 0.998. The synchronous correlation between baseline E2 and baseline sleep was 0.062 (95% CI, 0.012-0.112; *P* = .016). The path coefficient from baseline E2 to follow-up sleep was –0.054 (95% CI, –0.098 to –0.010; *P* = .016) and that of baseline sleep to follow-up E2 was –0.017 (95% CI, –0.067 to 0.034; *P* = .517). The synchronous correlation between baseline DHAS and baseline sleep was 0.006 (95% CI, –0.044 to 0.056; *P* = .806). The path coefficient from baseline DHAS to follow-up sleep was 0.025 (95% CI, –0.019 to 0.069; *P* = .273) and that of baseline sleep to follow-up DHAS was 0.042 (95% CI, 0.007-0.076; *P* = .017). These results indicated a unidirectional temporal relationship between (1) FSH, E2, and DHAS and (2) sleep, and the changes in FSH and E2 preceded the changes in sleep status score, while the change of DHAS was later than sleep. The path coefficients between T and sleep were not significant statistically, suggesting that there was no temporal relationship between T and sleep. Fitting parameters of these models all showed good fit to the data.

**Figure 3:**
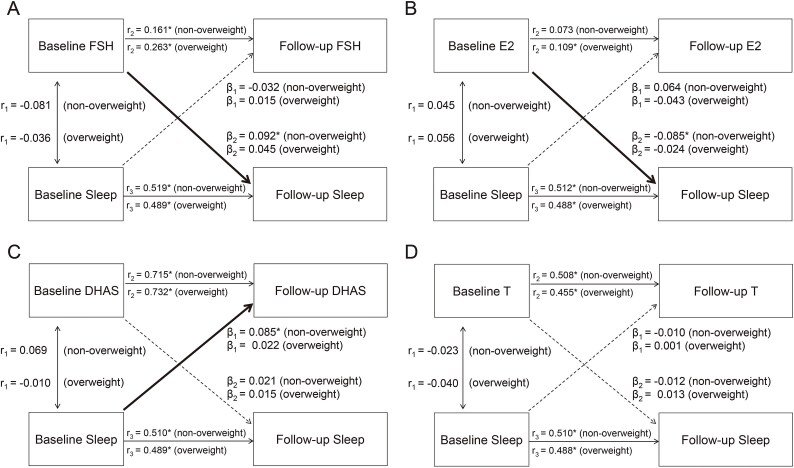
Cross-lagged path model between sex hormones and sleep status in nonoverweight and overweight groups, adjusted for age, race, income, status, HT use, smoking, and drinking. β_1_ and β_2_, cross-lagged path coefficients; *r*_1_, synchronous correlations; *r*_2_ and *r*_3_, tracking correlations. ^*^P < .05. DHAS, dehydroepiandrosterone sulfate; E2, estradiol; FSH, follicle-stimulating hormone; HT, hormone therapy; T, testosterone.

Stratified analyses in nonoverweight and overweight groups are depicted in Figure 3 and Table S3, with adjustment for age, race, income, menopausal status, HT use, smoking, and drinking. In nonoverweight participants (n = 729), the path coefficients from baseline FSH, E2, DHAS, and T to follow-up sleep were 0.092 (95% CI, 0.022-0.162; *P* = .010), –0.085 (95% CI, –0.156 to –0.015; *P* = .018), 0.021 (95% CI, –0.049 to 0.092; *P* = .556), and –0.012 (95% CI, –0.083 to 0.059; *P* = .741), respectively. The path coefficients from baseline sleep to follow-up FSH, E2, DHAS, and T were –0.032 (95% CI, –0.113 to 0.049; *P* = .443), 0.064 (95% CI, –0.018 to 0.146; *P* = .127), 0.085 (95% CI, 0.028 to 0.141; *P* = .003), and –0.010 (95% CI, –0.081 to 0.060; *P* = .776). The patterns of the temporal relationship between sex hormones and sleep in nonoverweight participants were the same as in the total sample, and the point estimates of the significant path coefficients were larger. By contrast, there was no clear temporal relationship in the overweight group (n = 1401).

**Figure 4:**
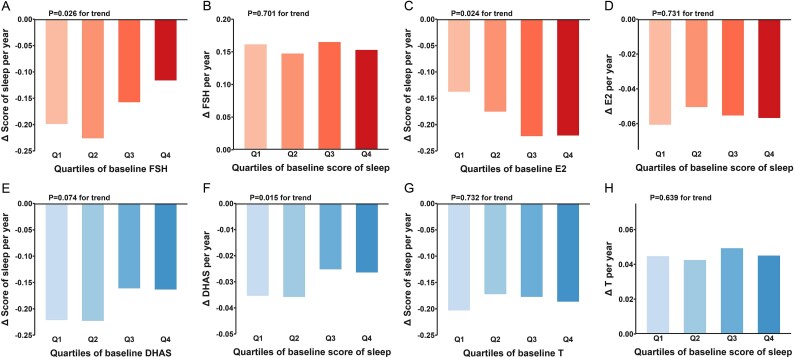
The yearly rates of change in sex hormones and sleep status by quartiles of their baseline values with adjustment for covariates. DHAS, dehydroepiandrosterone sulfate; E2, estradiol; FSH, follicle-stimulating hormone; T, testosterone.

After adjustment for VMSs, the path coefficients between sex hormone and sleep status were basically consistent with the main model, while the correlation between baseline FSH and E2 and baseline sleep was no longer significant (Figure S2, Table S4). Figure 4 illustrates the yearly rates of change in sex hormone and sleep by quartiles of their baseline values via general linear models to validate the results of cross-lagged path models in the total sample, with adjustment for the same covariates in the main model. The yearly rate of change in sleep during follow-up varied significantly across increasing quartiles of baseline FSH (*P* for trend = .026) and E2 (*P* for trend = .024); meanwhile, the yearly rate of change in DHAS showed a significant varying trend across quartiles of baseline sleep (*P* for trend = .015). These results were consistent with the temporal relationship of sex hormones and sleep shown in Figure 2. Results of the yearly rates of change in stratified analyses were also robust (Figures S3 and S4).

## Discussion

In this study, we have examined the temporal relationship between sex hormones and sleep status, using cross-lagged path analysis based on a longitudinal cohort of midlife women. There were distinct patterns in the unidirectional temporal relationships between (1) FSH, E2, and DHAS and (2) sleep. The change of FSH and E2 were prior to the sleep status score, while the change of DHAS was later than sleep. In contrast, there was no temporal relationship between T and sleep. Notably, the temporal relationship between sex hormones and sleep status was more significant in females who were nonoverweight.

At baseline, a negative relationship between FSH and sleep status was observed, which was consistent with previous studies.^13^^,^^14^ During menopause, significant fluctuations in FSH and other hormones are associated with sleep disturbances, including insomnia and fragmented sleep.^6^ A 10-year follow-up study based on 57 midlife women found that higher serum FSH level was associated with longer sleep latency (β = 0.45; 95% CI, 0.07-0.83) after controlling for BMI, vasomotor, and depressive symptoms.^7^ Similarly, Coborn et al reported that more awakenings were associated with higher FSH levels (β = 0.12; 95% CI, 0.02-0.22).^25^ This negative association at baseline might reflect the immediate impact of perimenopausal hormonal changes, where elevated FSH correlates with poorer sleep due to an increased prevalence of vasomotor symptoms, which are exacerbated by rising FSH levels and disrupt sleep.^8^^,^^26^ Additionally, our findings suggest that after controlling for VMSs, the negative relationship between FSH and sleep status at baseline was not significant. This also illustrates the importance of VMSs in this relationship.

However, cross-lagged panel analysis revealed a unidirectional positive association between baseline FSH with follow-up sleep quality. Participants with a higher FSH level at baseline may adapt to these hormonal changes faster and have a better sleep status at follow-up. The adaptation could involve stabilization of the endocrine system as women transition into postmenopause, where the initially disruptive effects of rising FSH diminish, leading to improved sleep quality.^27^ Burger et al noted that during perimenopause, the body’s response to a high FSH level is part of the natural hormonal shift leading to menopause.^28^ This pattern may be explained by the improved sleep status as women move further into the postmenopausal phase, when FSH levels stabilize and the body adjusts to the high level of FSH. Another study based on the SWAN cohort found that greater change of FSH was associated with poorer self-reported sleep quality, suggesting that women with higher baseline FSH may experience a lower rate of FSH change and therefore better sleep quality at follow-up.^14^ Similarly, a multicentric collaborative study found that in long-time sleepers, the levels of serum FSH were 20% higher than that in short-time sleepers (*P* = .008), and this association was independent of age and BMI.^9^ Nevertheless, the cross-sectional study design did not allow this multicentric study to figure out in which way the relationship between FSH level and sleep duration operated. Under a longitudinal framework, our study could indicate a clear temporal relationship that the change of FSH levels was earlier than that of sleep status. The underlying mechanisms of the longitudinal association need further exploration in a cohort study and laboratory investigation.

Conversely, our findings suggest a negative unidirectional relationship between baseline E2 and follow-up sleep status. The change of serum levels of E2 was prior to that of sleep status. A previous sleep substudy nested in SWAN based on small samples demonstrated that higher baseline E2 levels were related to higher Pittsburgh Sleep Quality Index score (representing poorer sleep quality), indicating that women with a higher E2 level at baseline may report poorer sleep quality 7 years later.^14^ Coborn et al found that E2 levels were associated with more awakenings (β = 0.14, *P* = .007) in the postmenopausal range.^25^ Evidence from premenopausal women in the late-luteal phase and animal studies also revealed that higher E2 levels were associated with more wakefulness after sleep onset.^29^^,^^30^

In basic and animal experiments, E2 administration at night has been shown to promote wakefulness in rodents.^31^^,^^32^ Estrogen receptors exist in various neural centers that regulate sleep, including the basal forebrain, hypothalamus, dorsal raphe nucleus, and locus coeruleus, as well as in the sleep-active ventrolateral preoptic area.^33-36^ Estrogen administration could alter neuronal activity in these sleep-regulating centers.^15^ This may be related to neuropeptide orexin A, whose expression in the hypothalamus and pituitary gland depends on E2 and is able to promote changes in wakefulness and menopause.^37^ Previous evidence showed that the levels of plasma orexin in postmenopausal women were 3 times higher than in premenopausal women, while plasma orexin levels in postmenopausal women receiving estrogen therapy were similar to those in premenopausal women.^38^ After controlling for hormone therapy, our analysis found a unidirectional temporal relationship from baseline E2 to follow-up sleep status. However, some studies have shown a positive correlation between E2 and sleep quality.^13^^,^^39^ We also observed a positive relationship between E2 and the sleep status score at baseline, but the positive relationship was nonsignificant after additional control for VMSs. As menopause approaches, increased severity of hot flashes is associated with a decline in E2, and hot flashes may mediate the association between E2 and sleep.^8^^,^^40^ Our findings suggest that women who have a lower FSH or higher E2 level at baseline in the transition of menopause are more likely to report sleep problems several years later. More attention should be paid to these individuals when developing policies for prevention and intervention of health problems related to menopause.

In contrast to the temporal sequence between estrogen and sleep status, there was a significant positive relationship between baseline sleep status and follow-up DHAS. Few studies have investigated the association between sleep parameters and DHAS. Jackowska et al found an inverse relationship between sleep disturbance and DHAS in men but not women, using data from the English Longitudinal Study of Ageing.^41^ Some studies have noted that interventions such as yoga, meditation, and mindfulness could increase DHAS levels significantly.^42-44^ Our findings suggest that changes in sleep quality might precede alterations in the serum levels of DHAS in midlife women, indicating that sleep disturbances may trigger changes in adrenal function. As an adrenal androgen, DHAS is involved in various physiologic processes, including sleep regulation. Poor sleep would dysregulate the hypothalamic-pituitary-adrenal axis, leading to altered cortisol and DHAS levels.^45^ Potential mechanisms of the positive effect of sleep quality on DHAS may involve stress and inflammation, which need further exploration.^46^

The absence of a significant temporal relationship between T and sleep in our study stands in contrast to some previous findings, particularly in men, where androgen levels have been linked to sleep quality.^47^^,^^48^ Liu et al found that short-term administration of high-dose T could shorten sleep and worsen sleep apnea in older men.^49^ Meanwhile, Wittert concluded that low T may affect overall sleep quality, which is improved by replacement doses, and that large doses of exogenous T and anabolic/androgenic steroid abuse were associated with abnormalities of sleep duration and architecture in men.^48^ However, Paul et al found that treatment with dihydrotestosterone did not affect sleep and wakefulness and that the effects of androgens appear to be gender specific.^50^ Our findings suggest that T may not play an important role in influencing sleep in midlife women, and the gender differences warrant further investigation.

Additionally, we found more pronounced temporal relationships between sex hormones and sleep in nonoverweight females. Previous studies have reported that obesity is related to low FSH levels in the transition to menopause.^51^^,^^52^ Estrogen deficiency may be an important predisposing factor for obesity in middle-aged women, which could worsen dysfunction of the metabolic system.^53^^,^^54^ Lower hormone levels in overweight women or, in other words, fewer hormonal variations may lead to the nonsignificant temporal relationship between hormones and sleep. Moreover, obesity results in the secretion of inflammatory factors that stimulate the expression of the aromatase enzyme, which is a product of the *CYP19* gene as well a member of the cytochrome P450 family.^55-57^ Aromatase is a rate-limiting enzyme that catalyzes the conversion of androgens to estrogens during steroidogenesis in the adipose tissue.^58^^,^^59^ Higher levels of aromatase receptor in obesity may regulate FSH, E2, and T levels and attenuate menopausal symptoms. Yet, obesity has been linked to depression and anxiety in menopausal women.^60^ The frequency of depression and anxiety disorder diagnoses is approximately double for obese women as compared with obese men.^61^^,^^62^ These psychological symptoms may have a more substantial impact on sleep quality than hormone changes. Our findings in overweight women could indicate that the effect of sex hormones on sleep may be overshadowed by metabolic dysregulation, anxiety, depression, and other symptoms, emphasizing the importance of considering metabolic and mental health in studies on hormonal regulation of sleep.

There are several strengths in the current study. This study used a longitudinal theoretical model to distinguish the temporal relationship between sex hormones and sleep status in midlife women. Evidence from this study could provide a scientific perspective to the health management of hormones and sleep in the transition to menopause. Nonetheless, several limitations should be acknowledged. First, although the temporal relationship is the first principle of causality inference, it is still not enough to clarify an underlying causal relationship in this observational study. Unknown confounders were not considered although we did adjust for possible covariates. Second, to obtain more general conclusions among middle-aged women and to ensure a sufficient sample size, we included postmenopausal women at baseline, which may introduce a little bias. However, the postmenopausal phase, while marking the end of the menstrual cycle, does not necessarily signal the end of all menopausal symptoms.^63^^,^^64^ We adjusted for menopausal status in all the models to mitigate the impact of menopausal status on our findings. Finally, our findings should be carefully extrapolated to other populations due to the particularity of our participants. Further research based on larger populations and experimental studies is needed to enhance the understanding of the temporal relationship between sex hormones and sleep.

In conclusion, there are different temporal relationships between sex hormone and sleep status in midlife women. Changes in estrogen such as FSH and E2 occurred earlier than the change of sleep, while the change of DHAS was later. In the management of hormones and sleep in middle-aged women, emphasis should be placed on the role of obesity and metabolism. Furthermore, topics focused on the relationship between estrogen and sleep should not be limited in baseline or cross-sectional associations, and evidence from longitudinal studies warrants more consideration.

## Supplementary Material

Revised_supplementary_Material_qfaf009
