# Supplementary material for: Different temporal relationship between sex hormones and sleep status in midlife women: a longitudinal cohort study
Source: Sex Med. 2025 Mar 2;13(1):qfaf009. doi: 10.1093/sexmed/qfaf009 (PMC11879248; doi:10.1093/sexmed/qfaf009)
Supplement: Revised_supplementary_Material_qfaf009 [file revised_supplementary_material_qfaf009.docx]

**Supplementary Material**

**Different temporal relationship between sex hormone and sleep status in perimenopausal women****: A longitudinal cohort study**

**Contents**

[**Table S1.** Encoding of sleep status-related variables 3](#_Toc177211709)

[**Table S2.** The cross-lagged path coefficients between sex hormone and sleep status 4](#_Toc177211710)

[**Table S3.** The cross-lagged path coefficients between sex hormone and sleep status in non-overweight and overweight groups 5](#_Toc177211711)

[**Table S4.** The cross-lagged path coefficients between sex hormone and sleep status further adjusted for VMS in the main model. 7](#_Toc177211712)

[**Figure S1.** Cross-lagged path model 8](#_Toc177211713)

[**Figure S2.** Cross-lagged path model between sex hormones and sleep status further adjusted for VMS in the main model. 9](#_Toc177211714)

[**Figure S3.** The yearly rates of change in sex hormones and sleep status by quartiles of their baseline values with adjustment for covariates in non-overweight group. 10](#_Toc177211715)

[**Figure S4.** The yearly rates of change in sex hormone and sleep status by quartiles of their baseline values with adjustment for covariates in overweight group. 11](#_Toc177211716)

**Table S1.** Encoding of sleep status-related variables

| Variables | question | Code |
| --- | --- | --- |
| RESTLES | I feel nervous and restless. | 1 (Almost Always)  2 (Often)  3 (Sometimes)  4 (Almost Never) |
| TRBLSLE | Did you have trouble falling asleep? (In the past two weeks) | 1 (Yes, 5 or more times a week)  2 (Yes, 3 or 4 times a week)  3 (Yes, 1 or 2 times a week)  4 (Yes, less than once a week)  5 (No, not in the past 2 weeks) |
| WAKEUP | Did you wake up several times a night? (In the past two weeks) | 1 (Yes, 5 or more times a week)  2 (Yes, 3 or 4 times a week)  3 (Yes, 1 or 2 times a week)  4 (Yes, less than once a week)  5 (No, not in the past 2 weeks) |
| WAKEARL | Did you wake up earlier than you had planned to, and were unable to fall asleep again? (In the past two weeks) | 1 (Yes, 5 or more times a week)  2 (Yes, 3 or 4 times a week)  3 (Yes, 1 or 2 times a week)  4 (Yes, less than once a week)  5 (No, not in the past 2 weeks) |
| SLEEPQL | During the past month, how would you rate your sleep quality overall? | 1 (Very bad)  2 (Fairly bad)  3 (Fairly good)  4 (Very good) |

**Table S2.** The cross-lagged path coefficients between sex hormone and sleep status

|  | Synchronous  Correlations (r_1_) | Path Coefficients | |  | Autocorrelation  Coefficients | |  | Goodness of  Model Fit | |
| --- | --- | --- | --- | --- | --- | --- | --- | --- | --- |
|  |  | β_1_ (sleep→hormone) | β_2_ (hormone→sleep) |  | sleep | hormone |  | RMR | CFI |
| FSH | -0.075*  (-0.125, -0.024) | 0.005  (-0.044, 0.054) | 0.054*  (0.010, 0.098) |  | 0.483*  (0.440, 0.527) | 0.201*  (0.152, 0.250) |  | 0.009 | 0.998 |
| E2 | 0.062*  (0.012, 0.112) | -0.017  (-0.067, 0.034) | -0.054*  (-0.098, -0.010) |  | 0.482*  (0.438, 0.526) | 0.061*  (0.011, 0.111) |  | 0.022 | 0.979 |
| DHAS | 0.006  (-0.044, 0.056) | 0.042*  (0.007, 0.076) | 0.025  (-0.019, 0.069) |  | 0.480*  (0.436, 0.524) | 0.726*  (0.692, 0.760) |  | <0.001 | 1.000 |
| T | -0.024  (-0.074, 0.026) | 0.013  (-0.031, 0.057) | 0.018  (-0.026, 0.062) |  | 0.480*  (0.436, 0.524) | 0.477*  (0.433, 0.521) |  | 0.003 | 1.000 |

FSH, follicle-stimulating hormone; E2, estradiol; DHAS, dehydroepiandrosterone sulfate; T, testosterone; RMR, root mean square residual; CFI, comparative fit index.

*, *P* < 0.05.

**Table S3.** The cross-lagged path coefficients between sex hormone and sleep status in non-overweight and overweight groups

|  | Synchronous  Correlations (r_1_) | Path Coefficients | |  | Autocorrelation  Coefficients | |  | Goodness of  Model Fit | | | |
| --- | --- | --- | --- | --- | --- | --- | --- | --- | --- | --- | --- |
|  |  | β_1_ (sleep→hormone) | β_2_ (hormone→sleep) |  | sleep | hormone |  | RMR | | CFI | |
| Non-overweight (N = 729) | | | | | | | | | | | |
| FSH | -0.081  (-0.163, 0.001) | -0.032  (-0.113, 0.049) | 0.092*  (0.022, 0.162) |  | 0.519*  (0.449, 0.589) | 0.161*  (0.080, 0.242) |  | 0.019 | | 0.990 | |
| E2 | 0.045  (-0.037, 0.127) | 0.064  (-0.018, 0.146) | -0.085*  (-0.156, -0.015) |  | 0.512*  (0.441, 0.582) | 0.073  (-0.009, 0.155) |  | 0.007 | | 1.000 | |
| DHAS | 0.069  (-0.012, 0.151) | 0.085*  (0.028, 0.141) | 0.021  (-0.049, 0.092) |  | 0.510*  (0.439, 0.580) | 0.715*  (0.658, 0.771) |  | 0.001 | | 1.000 | |
| T | -0.023  (-0.106, 0.059) | -0.010  (-0.081, 0.060) | -0.012  (-0.083, 0.059) |  | 0.510*  (0.439, 0.580) | 0.508*  (0.438, 0.579) |  | 0.006 | | 1.000 | |
| Overweight (N = 1401) | | | | | | | | | | | |
| FSH | -0.036  (-0.097, 0.026) | 0.015  (-0.044, 0.075) | 0.045  (-0.008, 0.099) |  | 0.489*  (0.436, 0.543) | 0.263*  (0.204, 0.322) | |  | 0.005 | | 1.000 |
| E2 | 0.056  (-0.005, 0.118) | -0.043  (-0.104, 0.018) | -0.024  (-0.078, 0.029) |  | 0.488*  (0.434, 0.542) | 0.109*  (0.048, 0.170) | |  | 0.009 | | 0.999 |
| DHAS | -0.010  (-0.071, 0.052) | 0.022  (-0.020, 0.064) | 0.015  (-0.039, 0.068) |  | 0.489*  (0.435, 0.542) | 0.732*  (0.690, 0.774) | |  | 0.005 | | 1.000 |
| T | -0.040  (-0.101, 0.022) | 0.001  (-0.054, 0.056) | 0.013  (-0.040, 0.067) |  | 0.488*  (0.435, 0.542) | 0.455*  (0.400, 0.510) | |  | 0.003 | | 1.000 |

FSH, follicle-stimulating hormone; E2, estradiol; DHAS, dehydroepiandrosterone sulfate; T, testosterone; RMR, root mean square residual; CFI, comparative fit index.

*, *P* < 0.05.

**Table S4.** The cross-lagged path coefficients between sex hormone and sleep status further adjusted for VMS in the main model.

|  | Synchronous  Correlations (r_1_) | Path Coefficients | |  | Autocorrelation  Coefficients | |  | Goodness of  Model Fit | |
| --- | --- | --- | --- | --- | --- | --- | --- | --- | --- |
|  |  | β_1_ (sleep→hormone) | β_2_ (hormone→sleep) |  | sleep | hormone |  | RMR | CFI |
| FSH | -0.029  (-0.080, 0.021) | 0.023  (-0.026, 0.072) | 0.047*  (0.003, 0.092) |  | 0.461*  (0.416, 0.506) | 0.224*  (0.175, 0.273) |  | 0.006 | 1.000 |
| E2 | 0.041  (-0.010, 0.091) | -0.045  (-0.095, 0.005) | -0.046*  (-0.091, -0.002) |  | 0.462*  (0.417, 0.506) | 0.081*  (0.031, 0.131) |  | 0.017 | 0.987 |
| DHAS | 0.007  (-0.043, 0.058) | 0.036*  (0.001, 0.070) | 0.026  (-0.018, 0.071) |  | 0.460*  (0.415, 0.504) | 0.725*  (0.690, 0.759) |  | 0.002 | 1.000 |
| T | -0.022  (-0.073, 0.028) | 0.006  (-0.038, 0.050) | 0.009  (-0.035, 0.054) |  | 0.460*  (0.415, 0.504) | 0.477*  (0.433, 0.522) |  | 0.003 | 1.000 |

VMS, vasomotor symptoms; FSH, follicle-stimulating hormone; E2, estradiol; DHAS, dehydroepiandrosterone sulfate; T, testosterone; RMR, root mean square residual; CFI, comparative fit index.

*, *P* < 0.05.


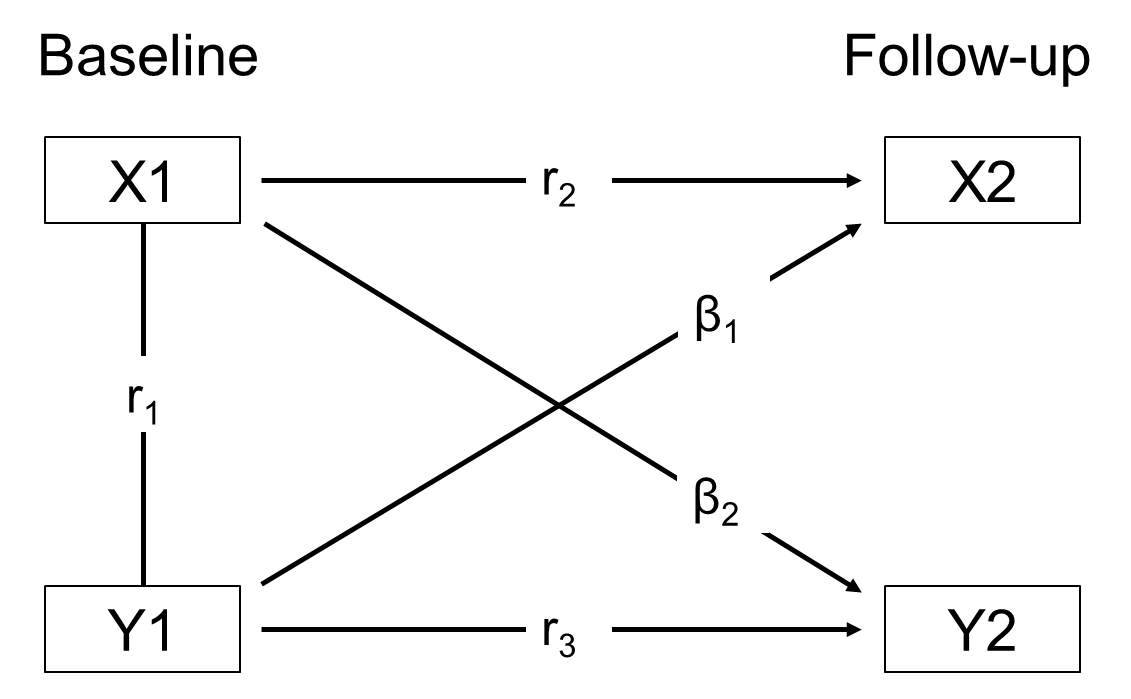


**Figure S1.** Cross-lagged path model

β_1_ and β_2,_ cross-lagged path coefficients; r_1,_ synchronous correlations; r_2_ and r_3_, tracking correlations.


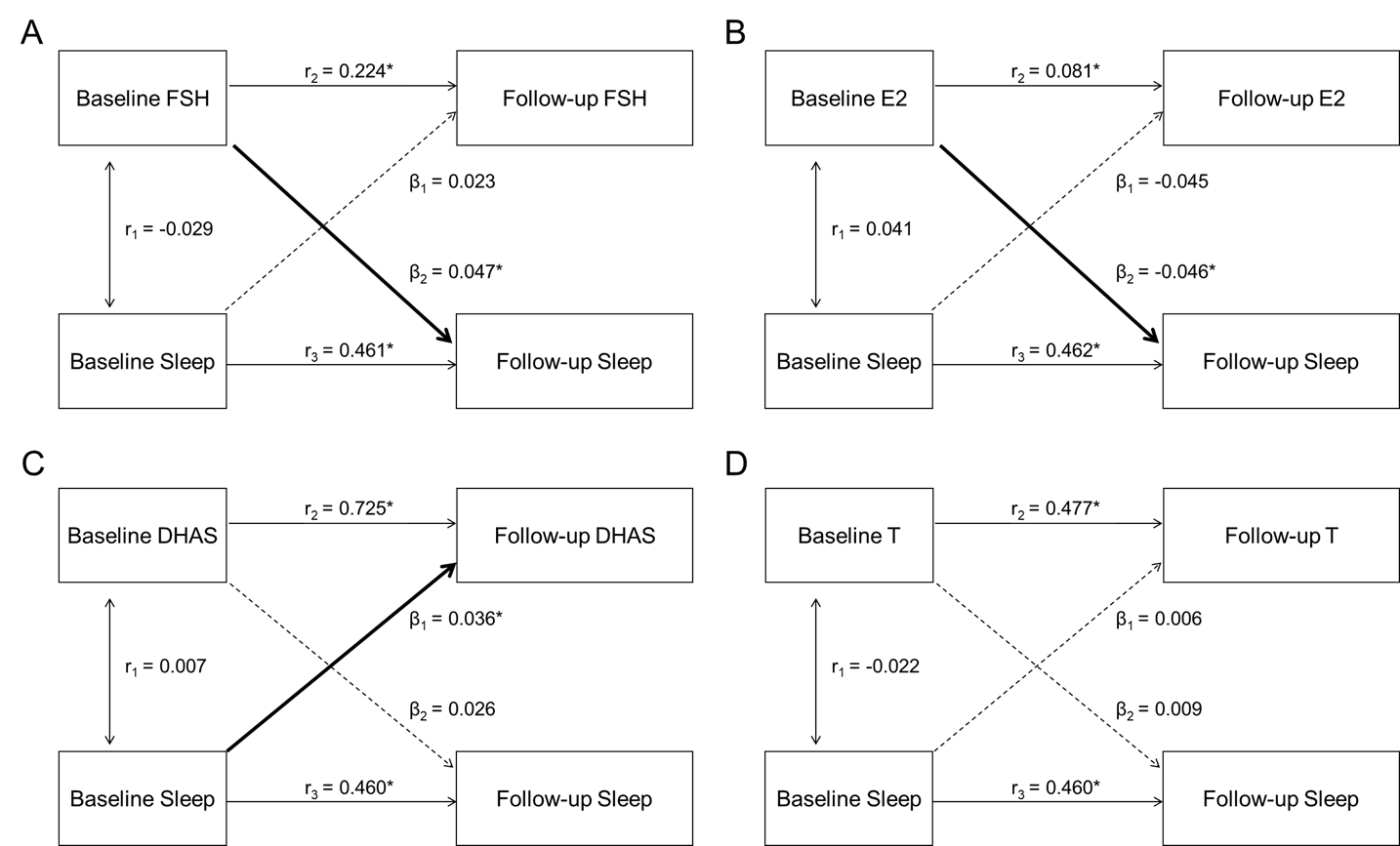


**Figure S2.** Cross-lagged path model between sex hormones and sleep status further adjusted for VMS in the main model.

VMS, vasomotor symptoms; FSH, follicle-stimulating hormone; E2, estradiol; DHAS, dehydroepiandro-sterone sulfate; T, testosterone.

β_1_ and β_2_, cross-lagged path coefficients; r_1_, synchronous correlations; r_2_ and r_3_, tracking correlations.

*, *P* < 0.05


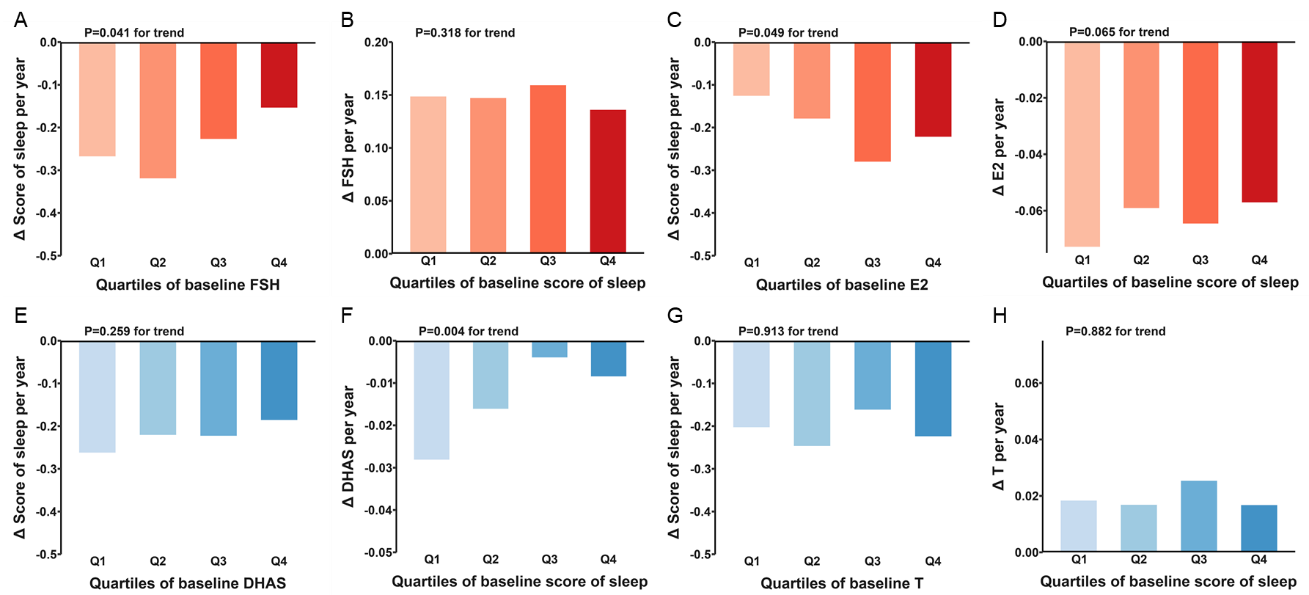


**Figure S3.** The yearly rates of change in sex hormones and sleep status by quartiles of their baseline values with adjustment for covariates in non-overweight group.

FSH, follicle-stimulating hormone; E2, estradiol; DHAS, dehydroepiandro-sterone sulfate; T, testosterone.


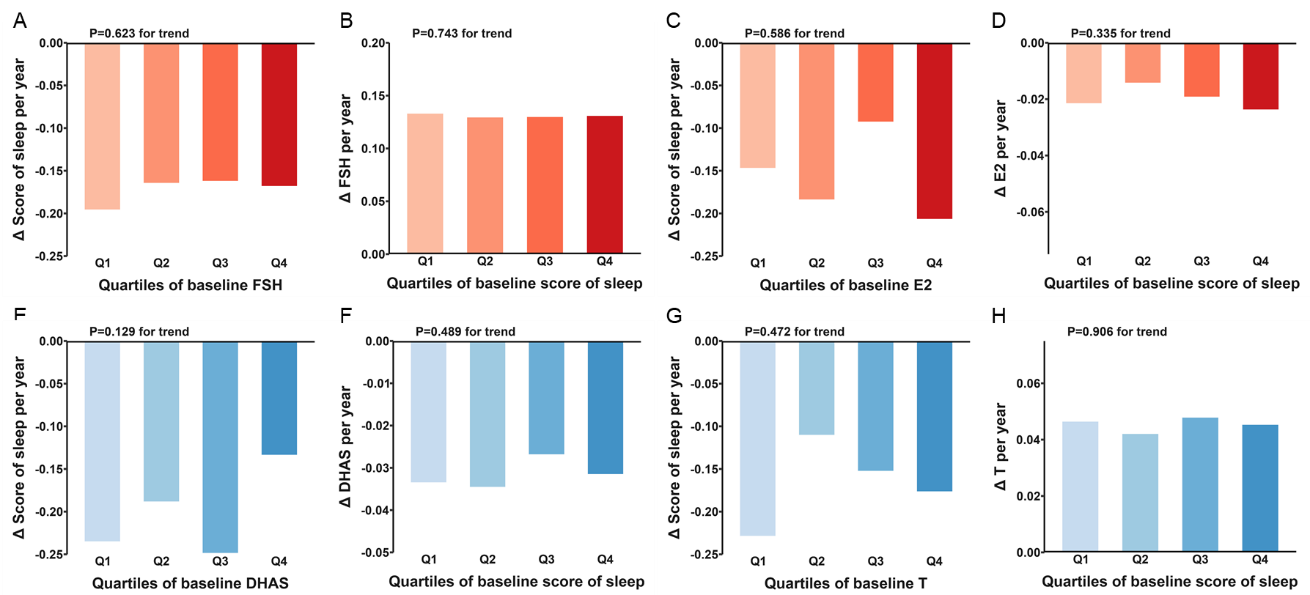


**Figure S4.** The yearly rates of change in sex hormone and sleep status by quartiles of their baseline values with adjustment for covariates in overweight group.

FSH, follicle-stimulating hormone; E2, estradiol; DHAS, dehydroepiandro-sterone sulfate; T, testosterone.
